# Association of plasma soluble CD14 level with asthma severity in adults: a case control study in China

**DOI:** 10.1186/s12931-019-0987-0

**Published:** 2019-01-28

**Authors:** Ting Zhou, Xiji Huang, Jixuan Ma, Yun Zhou, Yuewei Liu, Lili Xiao, Jing Yuan, Jungang Xie, Weihong Chen

**Affiliations:** 1 Department of Occupational and Environmental Health, School of Public Health, Medical College, Wuhan University of Science and Technology, Wuhan, 430065 Hubei China; 2 Hubei Province Key Laboratory of Occupational Hazard Identification and Control, Wuhan University of Science and Technology, Wuhan, 430065 Hubei China; 3 Department of Occupational and Environmental Health, School of Public Health, Tongji Medical College, Huazhong University of Science and Technology, 13 Hangkong Road, Wuhan, 430030 Hubei China; 4 Key Laboratory of Environment and Health, Ministry of Education & Ministry of Environmental Protection, and State Key Laboratory of Environmental Health (Incubating), School of Public Health, Tongji Medical College, Huazhong University of Science and Technology, Wuhan, 430030 Hubei China; 5 Hubei Center for Disease Control and Prevention, Wuhan, 430079 Hubei China; 6 Department of Respiratory and Critical Care Medicine, Tongji Hospital, Tongji Medical College, Huazhong University of Science and Technology, Wuhan, 430030 Hubei China

**Keywords:** sCD14, Adult asthma, Asthma severity

## Abstract

**Background:**

Soluble CD14 (sCD14) shedding from CD14 could regulate T lymphocyte activation and function, which has implicated in the pathogenesis of bronchial asthma. The level of sCD14 expression is obviously increased in asthmatic patients during acute asthma attacks. The objective of this study was to investigate the association between plasma sCD14 level and asthma severity in adults.

**Methods:**

The plasma sCD14 level in asthma patients (*n* = 910) and healthy controls (*n* = 881) was quantified by commercially available enzyme-linked immunosorbent assay (ELISA) kits. The asthma cases were subdivided into intermittent asthma (*n* = 537), mild (*n* = 246), moderate (*n* = 96) and severe (*n* = 31) persistent asthma patients. Association between plasma sCD14 level and asthma severity, lung function parameters as well as asthma symptoms and signs in adults were performed using multivariate logistic regression models.

**Results:**

We observed significant relationships of plasma sCD14 level with asthma severity, lung function parameters as well as asthma symptoms and signs in adults. After adjusting for multiple potential confounders, each one-unit increase in log sCD14 was significantly associated with 67, 82, 79 and 85% reduced ORs for intermittent asthma, mild, moderate and severe persistent asthma, respectively (all *P* < 0.0001). Compared with the participants of FEV_1_/FVC ≥75%, each one-unit increase in log sCD14 was significantly associated with a 37% decreased OR of FEV_1_/FVC < 75% (*P* < 0.0001). However, the adjusted odds ratios (ORs) of severe dyspnea, wheeze and cyanosis in asthma patients were 1.88, 1.46 and 2.20 for each one-unit increase in log sCD14, respectively. In addition, compared with health controls, the adjusted area under the curve (AUC) of sCD14 was 0.814 at a cut-off points of 0.53, and the sensitivity and specificity were 71.0 and 76.8% for predicting asthma in adults. And the adjusted AUC of sCD14 reached 0.786, 0.847, 0.887 and 0.917 in predicting intermittent asthma, mild, moderate and severe persistent asthma, respectively.

**Conclusions:**

Our results indicated that plasma sCD14 level is negatively associated with asthma severity, suggesting a protective role for sCD14 in the development of asthma in adults. And plasma sCD14 level might be a potential biomarker in prediction of asthma severity in adults.

**Electronic supplementary material:**

The online version of this article (10.1186/s12931-019-0987-0) contains supplementary material, which is available to authorized users.

## Introduction

Asthma, a chronic airway inflammatory disease, affects as many as 339.4 million individuals in the world, and its prevalence is estimated to be increasing rapidly in many developing countries [1]. Asthma with onset in adulthood is more likely to develop into persistent asthma, accompanied by decreased lung function at a slow rate, which has positive impacts on the quality of life during a lifetime [2–4]. The burden of asthma such as disability and premature death is mainly determined by the severity of asthma symptoms. However, possible mechanisms or biomarkers of asthma development or deterioration have not been well understood.

Airway inflammation is well known as a cardinal feature of asthma, characterized by infiltration of eosinophils, neutrophils, and CD4 T-helper cells into the airway submucosa [5, 6]. Recently, there is indeed evidence of monocytes/macrophages activation in bronchoalveolar space and peripheral blood in asthma exacerbations [7, 8]. Monocytes could differentiate into macrophages and dendritic cells, then regulate Th2-mediated immune response which contribute to the pathogenesis of asthma [9–11]. CD14, a marker of monocyte/macrophages activation, occurs in a membrane-bound form (mCD14) and a soluble form (sCD14) which has positive impact on the balance between Th1 and Th2 cytokines [12–14]. Especially, the sCD14 mainly derived from protease-dependent shedding of mCD14 and intracellular compartments plays an essential role in the regulation of T and B cells proliferation and activation [15–17]. Previous studies have found that the sCD14 level in asthmatic patients was significantly higher in asthma exacerbations than that at recovery period [18, 19]. But Martin reported that plasma sCD14 level was inversely correlated with asthma severity during acute asthma attacks in children [20]. Until now, few studies have focused on the exact association between sCD14 expression level and the severity of asthma in adults.

Therefore, we present this case-control study of 910 asthma cases and 881 healthy controls to investigate the association between plasma sCD14 level and asthma severity or clinic symptoms and signs of asthma in adults. To avoid potential influences from other confounders including age, gender, exercise, tobacco smoke exposure, and other environment factors which reported by published papers [21], we collected detailed information about personal physical characteristics, living habits, as well as exposure to environmental hazards. All above factors were adjusted in our statistical analysis.

## Material and methods

### Subjects

This case-control study included 910 asthma patients and 881 healthy controls that were older than 18 years and living in the communities at least 5 years in Wuhan city in China as described previously [22]. All asthma cases were outpatients and diagnosed by respiratory specialists from two big hospitals between February 2011 and January 2012 in Wuhan. The asthma cases were subdivided into intermittent (*n* = 537) and persistent (*n* = 373) asthma cases, and then the persistent asthma patients were further stratified into mild (*n* = 246), moderate (*n* = 96) and severe (*n* = 31) asthmatics. The healthy controls were selected from the same residential area as asthma patients, who were confirmed by the same physicians without any respiratory diseases and allergic diseases. All subjects have no infectious diseases, diabetes, hypertension and other cardiovascular and cerebrovascular diseases. This study protocol was approved by the Ethnics and Human Subject Committees of Tongji Medical School at the Huazhong University of Science and Technology (2011–17). Written informed consent was received from all participants.

### Data collection

Basic information was collected by trained investigators through face-to-face interviews using standard questionnaires, which included age, gender, occupational hazards exposure, family history of asthma, tobacco smoking and passive smoking status, alcohol drinking status, exercise, sleeping quality, and habits of keeping pets and planting flowers. Occupational hazards exposure was defined as whether individuals were exposed to industrial dust or toxicant in their workplaces. Smoking status was defined as whether individuals had been smoked more than one cigarette per day over the previous 6 months. Passive smoking status was defined as whether individuals had inhaled cigarette smoke caused by other people’s smoking for at least 15 min per day and more than one day a week. Non-smokers were considered as who smoked less than one cigarette per day. Drinking status was defined as whether individuals had drunk alcohol beverage more than once a week over the previous 6 months. Non-drinkers were considered as who drank less than once a week. Exercise was defined as regular exercise for more than 30 min at each time, at least once a week during the previous 6 months. Sleeping quality in the past six months was self-reported, and sleeping quality was classified into three categories: good, impaired and poor sleeping quality according to Pittsburgh Sleep Quality Index (PSQI) [23]. Good sleeping quality was defined as (1) falling asleep in 30 min, (2) hard to wake up at night, and (3) having a high spirit in daytime. Poor sleeping quality was defined as having sleep deprivation symptoms such as insomnia or taking hypnotics. Impaired sleeping quality was slightly impaired and indicated the status between good and poor sleeping quality, which was defined as meeting one of the following criteria: (1) difficult to fall asleep (≥ 3 times per week); (2) easy to wake up at night (≥ 3 times per night); and (3) frequent nightmares at night (≥ 3 times per week).

Standing height and body weight were measured when individuals were standing with light indoor clothing and without shoes. Body mass index (BMI) was calculated as weight in kilograms divided by the square of height in meters.

### Lung function test

All participants were tested for lung function in the sitting position with a nose clip after normal breathing at least 5 min by an electronic spirometer (Chestgraph HI-101, CHEST Ltd., Tokyo, Japan) and required to accomplish three satisfactory curves. The optimal data were selected for analyses according to the recommendations of the American Thoracic Society guidelines [24]. The results were expressed as expiratory volume (L) and percentage of the predicted values. The percentage of the predicted values of individuals were calculated by adjusting for gender, age and height. Forced vital capacity (FVC), percent of predicted FVC (%PRED FVC), forced expiratory volume in one second (FEV_1_), percent of predicted FEV_1_ (%PRED FEV_1_), FEV_1_/FVC, and peak expiratory flow (PEF) were obtained. It is suggested that the FEV_1_/FVC ratio is normally more than 0.75–0.80 in adults [25], so we categorized the FEV_1_/FVC ratio into two categorical variables by FEV_1_/FVC ≥75% (normal) and < 75% (abnormal).

### Clinical characteristics of asthma

The clinical symptoms and signs of asthma patients were also collected by the doctor’s inquiry and physical examination at the time of admission. Cyanosis is a clinical manifestation of bluish discoloration of the skin and mucosa caused by the presence of deoxygenated hemoglobin in the circulation. The clinical rating of dyspnea was categorized as five grades (Grade 0 to 4) according to the modified Medical Research Council (mMRC) dyspnea scale [26, 27]. The grading criteria are as followings: Grade 0: no breathlessness except on strenuous exercise; Grade 1: shortness of breath when hurrying on the level or walking up a slight hill; Grade 2: walking slower than people of same age on the level because of breathlessness or has to stop to catch breath when walking at their own pace on the level; Grade 3: stopping for breath after walking ∼100 m or after few minutes on the level; and Grade 4: too breathless to leave the house, or breathless when dressing or talking. Asthma patients were diagnosed by the presence of asthma symptoms and spirometry demonstrating airway hyperresponsiveness (Provoking Dose 20 (PD20) to methacholine < 2.5 mg) and/or bronchodilator responsiveness (an increase of FEV_1_ more than 12% and 200 ml after inhaling 200 μg salbutamol) according to Global Initiative for Asthma guidelines [25]. The severity of asthma was evaluated by frequency of asthma symptoms during day and night and the level of lung function parameters. The severity of asthma was classified into four grades: intermittent asthma (Grade 1), mild (Grade 2), moderate (Grade 3) and severe (Grade 4) persistent asthma based on Guidelines for Prevention and Treatment of Bronchial Asthma in China (2016 version), which refers to Global Initiative for Asthma guidelines [25]. The definitions of severity scale of asthma patients were shown in Additional file 1 Table S1.

### Plasma samples and sCD14 level determination

Approximately 5 mL peripheral blood samples were collected in a tube with Ethylenediaminetetraacetic acid (EDTA) for all participants. The blood samples of asthma patients and healthy controls were obtained within 24 h of admission or questionnaire survey, respectively. All plasma samples were separated by centrifugation at 1500 rpm for 10 min at room temperature and stored at − 80°C. The sCD14 level in plasma samples was determined by using enzyme-linked immunosorbent assay (ELISA) kits (R&D Systems, Minneapolis, USA) according to the manufacturer’s instructions, with the sensitivity of 125 pg/ml and assay range of 250.0–16,000 pg/ml.

### Statistical analysis

Socio-demographic and lifestyle characteristics distributions were compared by Chi-square test or Student's t-test between healthy controls and asthma patients. Plasma sCD14 level was log-transformed due to right-skewed distribution and expressed as medians with interquartile ranges (IQRs). Differences on the basic characteristics distributions by asthma severity were tested by using Mantel-Haenszel Chi-square test for categorical variables and linear regression for continuous variables. The differences of clinical symptoms and signs were also analyzed by Mantel-Haenszel Chi-square test in asthma patients. The associations of plasma sCD14 level with asthma severity, lung function parameters as well as clinical symptoms and signs of asthma in adults were assessed by multivariate logistic regression models, with adjustment for multiple potential confounders including age, gender, BMI, smoking and passive smoking status, alcohol drinking status, exercise, family history of asthma, keeping pets, planting flowers, sleeping quality. The relationships were quantified by using adjusted odds ratios (ORs) and 95% confidence intervals (CIs) of asthma severity, FEV_1_/FVC ratio and asthma symptoms and signs by using plasma sCD14 as continuous and categorical variables.

In addition, receiver operating characteristic (ROC) curves and the area under the curve (AUC) were used to assess the sensitivity and specificity of plasma sCD14 level as a novel biomarker for the prediction of asthma severity in adults. An optimal cut-off value was required for ROC curve to define the distinguishing power, which would represent the highest sum of sensitivity and specificity when the Youden index (Youden index = sensitivity + specificity − 1) reaches the maximum value. The *P* values < 0.05 were considered statistically significant, and SAS statistical software, version 9.3 (SAS Institute Inc., Cary, North Carolina) was used for the statistical analyses.

## Results

### Basic characteristics

Table 1 summarizes the sociodemographic data, lifestyle habits, lung function and plasma sCD14 level of the population studied. The age of 1791 participants ranged from 18 to 86 years, and mean age of persistent asthma patients (43.82 ± 13.57 years) was younger than that of intermittent asthma cases (46.91 ± 14.36 years) and healthy controls (46.63 ± 14.40 years). The percentages of asthma family history and poor sleeping quality in asthma patients were significantly higher than that in healthy controls (*P* < 0.0001) Compared with health controls, the percentages of exercise, current smoking, passive smoking, and current alcohol drinking status were significantly lower in asthma patients (*P* < 0.0001). No differences were found in age, BMI, as well as the percentages of female, occupational hazards exposure, planting flowers and keeping pets between healthy controls and asthma patients. However, age, the percentages of exercise, planting flowers and good sleeping quality were significantly decreased with increasing severity of asthma (*P* < 0.05) whereas the percentage of keeping pets was significantly elevated with increasing severity of asthma (*P* < 0.05).

**Table 1:** The basic characteristics of the study population

Variables	Control (*n* = 881)	All asthmatics (*n* = 910)	*P* value	Intermittent asthmatics (*n* = 537)	Persistent asthmatics (n = 373)			*P*_*trend*_
					Mild (*n* = 246)	Moderate (*n* = 96)	Severe (*n* = 31)	
Age (years, Mean ± SD)	46.63 ± 14.40	45.64 ± 14.11	0.1424	46.91 ± 14.36	43.76 ± 14.05	44.31 ± 12.31	42.71 ± 13.77	0.0040
Female (yes, %)	522 (59.25)	543 (59.67)	0.8565	320 (59.59)	148 (60.16)	60 (62.50)	15 (48.39)	0.7487
Occupational hazards exposure (yes, %)	306 (34.73)	337 (37.03)	0.3105	205 (38.18)	82 (33.33)	38 (39.58)	12 (38.71)	0.8230
Family history of asthma (yes, %)	5 (0.57)	100 (10.99)	< 0.0001	61 (11.36)	19 (7.72)	15 (15.63)	5 (16.13)	0.4609
Exercise (yes, %)	417 (47.33)	252 (27.69)	< 0.0001	176 (32.77)	59 (23.98)	11 (11.46)	6 (19.35)	< 0.0001
Keeping pets (yes, %)	132 (14.98)	153 (16.81)	0.2898	86 (16.01)	39 (15.85)	14 (14.58)	14 (45.16)	0.0313
Planting flowers (yes, %)	178 (20.20)	192 (21.10)	0.6401	123 (22.91)	53 (21.54)	10 (10.42)	6 (19.35)	0.0353
Smoking status (N, %)			< 0.0001					0.1750
No smoking	647 (73.44)	718 (78.90)		427 (79.52)	193 (78.46)	77 (80.21)	21 (67.74)	
Former smoking	22 (2.50)	74 (8.13)		46 (8.57)	20 (8.13)	6 (6.25)	2 (6.45)	
Current smoking	212 (24.06)	118 (12.97)		64 (11.92)	33 (13.41)	13 (13.54)	8 (25.81)	
Passive smoking (yes, %)	302 (34.28)	217 (23.85)	< 0.0001	143 (26.63)	49 (19.92)	23 (23.96)	2 (6.45)	0.5931
Drinking (N, %)			< 0.0001					0.6185
No drinking	671 (76.16)	767 (84.29)		457 (85.10)	206 (83.74)	78 (81.25)	26 (83.87)	
Former drinking	18 (2.04)	49 (5.38)		26 (4.84)	12 (4.88)	9 (9.38)	2 (6.45)	
Current drinking	192 (21.79)	94 (10.33)		54 (10.06)	28 (11.38)	9 (9.38)	3 (9.68)	
Sleeping quality (N, %)			< 0.0001					0.0015
Good	538 (61.07)	250 (27.47)		172 (32.03)	59 (23.98)	13 (13.54)	6 (19.35)	
Impaired	278 (31.56)	442 (48.57)		243 (45.25)	127 (51.63)	55 (57.29)	17 (54.84)	
Poor	65 (7.38)	218 (23.96)		122 (22.72)	60 (24.39)	28 (29.17)	8 (25.81)	
BMI (kg/m^2^, Mean ± SD)	23.22 ± 3.36	23.03 ± 3.48	0.2261	23.14 ± 3.54	23.02 ± 3.45	22.86 ± 3.39	21.63 ± 2.56	0.0523
Lung function parameters								
FVC (L, Mean ± SD)	3.11 ± 0.88	2.91 ± 1.03	< 0.0001	2.84 ± 1.01	3.00 ± 1.02	3.07 ± 1.05	2.98 ± 1.20	0.0197
FEV_1_ (L, Mean ± SD)	2.61 ± 0.69	2.11 ± 0.84	< 0.0001	2.09 ± 0.82	2.20 ± 0.84	2.04 ± 0.85	1.94 ± 1.02	0.7273
% PRED FVC, % (Mean ± SD)	102.77 ± 23.75	88.65 ± 23.12	< 0.0001	87.77 ± 22.87	90.39 ± 22.65	90.60 ± 23.48	83.67 ± 29.19	0.5332
% PRED FEV_1_, % (Mean ± SD)	98.05 ± 24.68	77.82 ± 26.19	< 0.0001	77.86 ± 25.63	80.12 ± 24.73	75.42 ± 30.28	66.22 ± 30.69	0.1292
FEV_1_/FVC (%)	84.73 ± 8.18	72.52 ± 15.18	< 0.0001	73.88 ± 15.02	73.08 ± 13.39	66.26 ± 16.48	63.79 ± 19.68	< 0.0001
PEF	5.52 ± 2.13	4.63 ± 2.08	< 0.0001	4.56 ± 2.08	4.79 ± 2.09	4.70 ± 2.03	4.27 ± 2.09	0.6808
sCD14 (ng/mL, Median, 25th~75th)	708.21 (499.91, 967.66)	500.47 (334.59, 744.65)	< 0.0001	555.99 (364.86, 791.24)	421.02 (296.82, 671.69)	460.53 (315.78, 677.69)	398.56 (258.49, 534.96)	< 0.0001

Lung function parameters including %PRED FVC, FEV_1_, %PRED FEV_1_, FEV_1_/FVC and PEF in asthma cases were significantly lower in comparison with healthy controls (*P* < 0.0001). Without adjustment for confounders, the median of plasma sCD14 level was significantly decreased in asthma patients when compared with health controls (*P* < 0.0001). In addition, the levels of FEV_1_/FVC and plasma sCD14 expression were significantly declined with increasing severity of asthma (*P* < 0.0001).

### Clinical symptoms and signs of asthma patients

As shown in Table 2, the percentage of severe dyspnea was significantly elevated with the increasing severity of asthma (*P* < 0.0001). No significant differences were found in the proportions of cough, sputum status, wheeze and cyanosis across the increasing severity of asthma in all asthma patients.

**Table 2:** Characteristics of clinical symptoms and signs in asthma patients

Clinical symptoms and signs	Intermittent asthma (*n* = 537)	Persistent asthma (*n* = 373)			*P*_*trend*_
		Mild (*n* = 246)	Moderate (*n* = 96)	Severe (*n* = 31)	
Cough status (N, %)					0.0520
No	115 (21.42)	39 (15.85)	19 (19.79)	10 (32.26)	
Occasional^a^	204 (37.99)	84 (34.15)	23 (23.96)	6 (19.35)	
Chronic^b^	218 (40.60)	123 (50.00)	54 (56.25)	15 (48.39)	
Sputum status (N, %)					0.1757
No	178 (33.15)	77 (31.30)	27 (28.13)	10 (32.26)	
Occasional^a^	199 (37.06)	86 (34.96)	30 (31.25)	12 (38.71)	
Chronic^b^	122 (22.72)	71 (28.86)	30 (31.25)	6 (19.35)	
Purulent	38 (7.08)	12 (4.88)	9 (9.38)	3 (9.68)	
Dyspnea (N, %)^c^					< 0.0001
No	179 (33.33)	73 (29.67)	15 (15.63)	7 (22.58)	
Mild	175 (32.59)	81 (32.93)	16 (16.67)	3 (9.68)	
Moderate	129 (24.02)	64 (26.02)	44 (45.83)	7 (22.58)	
Severe	54 (10.06)	28 (11.38)	21 (21.88)	14 (45.16)	
Wheeze (yes, %)	149 (27.75)	62 (25.20)	29 (30.21)	17 (54.84)	0.0564
Cyanosis (yes, %)	33 (6.15)	12 (4.88)	8 (8.33)	4 (12.90)	0.2561

^a^represents no more than three times a week; ^b^represents last for more than two months; ^c^indicated the severity of dyspnea including mild (Grade 0 and 1); moderate (Grade 2); severe (Grade 3 and 4)

### Association of plasma sCD14 level and severity of adult asthma

The association between plasma sCD14 level and the severity of adult asthma is shown in Table 3. After adjusting for multiple potential confounders, each one-unit increase in log sCD14 was statistically associated with a 73% reduction in the risk (OR) of adult asthma. In categorical analysis, we observed that increased plasma sCD14 level was significantly associated with decreased risk of adult asthma in a dose-dependent manner (*P*_trend_ < 0.0001). Compared with the lowest quartile, the adjusted ORs (95%CI) for adult asthma were 0.45 (0.32, 0.62), 0.29 (0.21, 0.40) and 0.19 (0.14, 0.26) across the increasing quartiles of sCD14.

**Table 3:** Association of plasma sCD14 level with asthma severity in adults

Asthma severity	Q1	Q2	Q3	Q4	*LogCD14*	*P*_*trend*_
	< 398.54	398.54~607.51	607.51~855.01	> 855.01		
Asthma/control	327/119	241/208	197/251	145/303		
Adjusted OR (95% CI)	reference	0.45 (0.32, 0.62)	0.29 (0.21, 0.40)	0.19 (0.14, 0.26)	0.27 (0.22, 0.34)	< 0.0001
Intermittent/control	165/119	136/208	135/251	101/303		
Adjusted OR (95% CI)	reference	0.48 (0.33, 0.68)	0.38 (0.27, 0.54)	0.25 (0.17, 0.35)	0.33 (0.26, 0.43)	< 0.0001
Mild/control	110/119	66/208	39/251	31/303		
Adjusted OR (95% CI)	reference	0.38 (0.25, 0.58)	0.17 (0.11, 0.26)	0.12 (0.07, 0.20)	0.18 (0.14, 0.25)	< 0.0001
Moderate/control	37/119	28/208	19/251	12/303		
Adjusted OR (95% CI)	reference	0.51 (0.28, 0.91)	0.25 (0.13, 0.47)	0.14 (0.07, 0.28)	0.21 (0.14, 0.32)	< 0.0001
Severe/control	15/119	11/208	4/251	1/303		
Adjusted OR (95% CI)	reference	0.61 (0.25, 1.46)	0.14 (0.04, 0.45)	0.03 (0.003, 0.22)	0.15 (0.08, 0.28)	< 0.0001

Estimates were adjusted for age, sex, BMI, smoking status, passive smoking status, alcohol consumption, physical activity, family history of asthma, keeping pets, planting flowers, sleeping quality

Furthermore, with adjustment for multiple potential confounders, each one-unit increase in log sCD14 was significantly associated with a 67, 82, 79 and 85% reduced ORs for intermittent asthma, mild, moderate and severe persistent asthma, respectively. Significantly negative relationships were also observed between plasma sCD14 level and severity of adult asthma by categorical analysis (*P*_trend_ < 0.0001). Compared with the lowest quartile of sCD14, the adjusted ORs (95%CI) for patients with intermittent asthma, mild, moderate and severe persistent asthma in adults were 0.25 (0.17, 0.35), 0.12 (0.07, 0.20), 0.14 (0.07, 0.28) and 0.03 (0.003, 0.22) in the highest quartile of sCD14, respectively. To avoid the influence of complications of elderly asthma on these results, we recalculated and got the similar results when deleted 325 participants (164 asthma patients and 161 controls).

### Association between plasma sCD14 level and lung function parameters

In Table 4, compared with the participants of FEV_1_/FVC ≥75%, each one-unit increase in log sCD14 was significantly associated with a 37% decreased OR of FEV_1_/FVC < 75% in continuous analysis after adjusting for multiple potential confounders. We also found that upward trend of plasma sCD14 level is statistically associated with declined the probability of FEV_1_/FVC < 75% by categorical analysis (*P*_trend_ < 0.0001). Compared with the lowest quartile, the adjusted OR (95%CI) of participants with FEV_1_/FVC < 75% were 0.83 (0.62, 1.11), 0.53 (0.40, 0.72) and 0.59 (0.44, 0.79) from the second quartile to the fourth quartile of sCD14.

**Table 4:** Association between plasma sCD14 level and Lung Function Parameter (FEV_1_/FVC, %)

FEV_1_/FVC (%)	Q1	Q2	Q3	Q4	*LogCD14*	*P*_*trend*_
	< 398.54	398.54~607.51	607.51~855.01	> 855.01		
< 75% / ≥75%	194/252	157/292	116/332	116/332		
OR (95% CI)	reference	0.70 (0.53, 0.91)	0.45 (0.34, 0.60)	0.45 (0.34, 0.60)	0.54 (0.45, 0.64)	< 0.0001
Adjusted OR (95% CI)	reference	0.83 (0.62, 1.11)	0.53 (0.40, 0.72)	0.59 (0.44, 0.79)	0.63 (0.53, 0.76)	< 0.0001

Estimates were adjusted for age, sex, BMI, smoking status, passive smoking status, alcohol consumption, physical activity, family history of asthma, keeping pets, planting flowers, sleeping quality

### Association of plasma sCD14 level with clinical symptoms and signs in asthma patients

Table 5 presents the results of association between plasma sCD14 level and clinical symptoms and signs in asthma patients, using both continuous and categorical analysis and adjusting for multiple potential confounders. In continuous analysis, the adjusted ORs (95%CI) of severe dyspnea, wheeze and cyanosis in asthma patients were 1.88, 1.46 and 2.20 for each one-unit increase in log sCD14, respectively. The categorical analysis also revealed significantly positive relationships between plasma sCD14 level and increased risk of severe dyspnea, wheeze and cyanosis in a dose-dependent manner (all *P*_trend_ < 0.05). The adjusted ORs (95%CI) for asthma patients with severe dyspnea, wheeze and cyanosis in the highest quartile of sCD14 were 2.33 (1.21, 4.48), 1.77 (1.15, 2.72) and 2.68 (1.13, 6.37) when compared with those in the lowest quartile, respectively. No significant association was observed between plasma sCD14 level and the other clinical symptoms and signs of asthma such as cough and sputum status.

**Table 5:** Association of plasma sCD14 level with clinical symptoms and signs in asthma patients

Clinical symptoms and signs		Q1	Q2	Q3	Q4	*LogCD14*	*P*_*trend*_
		< 334.59	334.59–500.47	500.47–744.65	> 744.65		
Symptoms							
Cough status	Occasional	reference	0.82 (0.48, 1.39)	1.14 (0.69, 1.89)	1.60 (0.85, 3.02)	1.16 (0.84, 1.62)	0.1432
	Chronic	reference	0.69 (0.42, 1.13)	0.80 (0.49, 1.31)	1.53 (0.83, 2.80)	0.93 (0.69, 1.27)	0.4761
Sputum status	Occasional	reference	1.22 (0.77, 1.94)	1.00 (0.63, 1.58)	1.33 (0.83, 2.13)	1.10 (0.83, 1.46)	0.4091
	Chronic	reference	0.83 (0.51, 1.34)	0.55 (0.34, 0.91)	0.88 (0.54, 1.45)	0.76 (0.57, 1.03)	0.4333
	Purulent	reference	1.44 (0.59, 3.51)	1.74 (0.74, 4.06)	2.09 (0.89, 4.95)	1.59 (0.94, 2.67)	0.1150
Dyspnea	Mild	reference	0.94 (0.59, 1.51)	0.99 (0.61, 1.60)	0.87 (0.53, 1.44)	0.96 (0.72, 1.29)	0.6831
	Moderate	reference	0.96 (0.58, 1.57)	0.92 (0.55, 1.53)	1.08 (0.65, 1.77)	1.09 (0.80, 1.49)	0.7262
	Severe	reference	1.09 (0.54, 2.21)	1.98 (1.02, 3.86)	2.33 (1.21, 4.48)	1.88 (1.24, 2.85)	0.0030
Sign							
Wheeze		reference	1.19 (0.77, 1.85)	1.56 (1.01, 2.41)	1.77 (1.15, 2.72)	1.46 (1.12. 1.92)	0.0055
Cyanosis		reference	1.38 (0.53, 3.59)	2.99 (1.26, 7.08)	2.68 (1.13, 6.37)	2.20 (1.29, 3.73)	0.0151

Estimates were adjusted for age, sex, BMI, smoking status, passive smoking status, alcohol consumption, physical activity, family history of asthma, keeping pets, planting flowers, sleeping quality

### Predictive effect of plasma sCD14 on different severity of asthma in adults

**Fig. 1:**
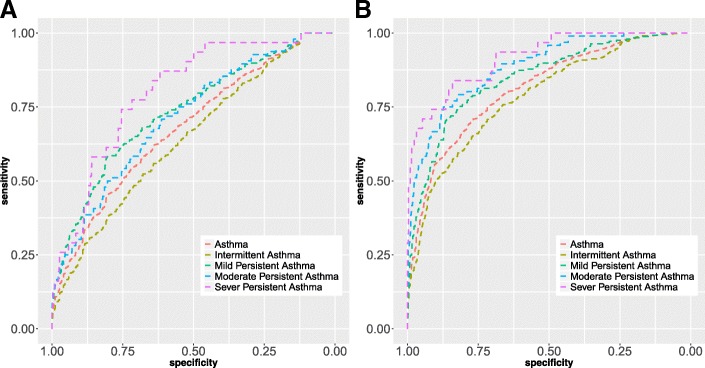
Receiver operating characteristic (ROC) curves for the severity of adult asthma by plasma sCD14 level. **a** represents unadjusted ROC curves. **b** indicates the ROC curves adjusted by age, sex, BMI, smoking status, passive smoking status, alcohol consumption, physical activity, family history of asthma, keeping pets, planting flowers, sleeping quality

The predictive role of plasma sCD14 level on adult asthma and its severity were shown in Table 6 and Fig. 1. Compared with health controls, the adjusted AUC (95%CI) of sCD14 was 0.814 (0.795, 0.833) at a cut-off value of 0.53 with a sensitivity of 71.0% and specificity of 76.8% for adult asthma. The adjusted AUC (95%CI) of sCD14 reached 0.786, 0.847, 0.887, 0.917 at cut-off points of 0.37, 0.30, 0.13, 0.03, and the sensitivity and specificity were 70.8, 70.7, 75.0, 83.9% and 71.9, 86.3, 87.3, 84.1% for predicting intermittent asthma, mild, moderate and severe persistent asthma, respectively. The results indicated that plasma sCD14 might be a potential biomarker in prediction of asthma severity in adults.

**Table 6:** Receiver operating characteristic (ROC) of sCD14 in plasma for asthma severity in adults

Asthma severity	AUC (95%CI)	*P* value	Cut off point	Sensitivity	Specificity
Asthma					
unadjusted	0.681(0.656, 0.705)	< 0.001	0.56	58.1%	68.2%
adjusted	0.814 (0.795, 0.833)	< 0.001	0.53	71.0%	76.8%
Intermittent asthma					
unadjusted	0.642 (0.613, 0.672)	< 0.001	0.43	52.3%	67.9%
adjusted	0.786 (0.761, 0.810)	< 0.001	0.37	70.8%	71.9%
Mild persistent asthma					
unadjusted	0.735(0.699, 0.772)	< 0.001	0.31	58.1%	80.6%
adjusted	0.847 (0.819, 0.876)	< 0.001	0.30	70.7%	86.3%
Moderate persistent asthma					
unadjusted	0.715 (0.661, 0.769)	< 0.001	0.10	70.8%	61.3%
adjusted	0.887 (0.852 0.921)	< 0.001	0.13	75.0%	87.3%
Severe persistent asthma					
unadjusted	0.802 (0.731, 0.873)	< 0.001	0.05	74.2%	75.5%
adjusted	0.917 (0.867, 0967)	< 0.001	0.03	83.9%	84.1%

Estimates were adjusted for age, sex, BMI, smoking status, passive smoking status, alcohol consumption, physical activity, family history of asthma, keeping pets, planting flowers, sleeping quality

## Discussion

In the present study, we found significantly negative relationship of plasma sCD14 level with asthma severity and the probability of FEV_1_/FVC < 75% in adults. It is suggested that plasma sCD14 level might have a predictive effect on the severity of asthma in adults. But there was significantly positive association of plasma sCD14 level with clinical symptoms and signs such as severe dyspnea, wheeze and cyanosis in adult asthma patients.

Asthma is a complex respiratory disorder, mainly characterized by recurrent episodes of reversible airflow obstruction, bronchial hyperresponsiveness and airway inflammation [28]. Acute attack asthma is usually accompanied by typical clinical symptoms and signs such as an episode of cough, breathlessness, dyspnea, wheeze and even cyanosis. In our results, we found that elevated plasma sCD14 level was significantly associated with symptoms and signs of asthma including severe dyspnea, wheeze and cyanosis at the time of admission. It is indicated that monocytes and macrophages could play an important role in the inflammatory process of acute asthma [29, 30]. The myelomonocytic differentiation antigen CD14 is a key component of innate immune system, which is mainly expressed on monocytes and various tissue macrophages, and in smaller amounts on granulocytes and B-lymphocytes [31]. It exists in membrane-bound form (mCD14) and soluble form (sCD14), and serves as a multifunctional receptor for lipopolysaccharides (LPS), endotoxins and other bacterial wall components [32, 33]. The binding of LPS to sCD14 could induce macrophage and bronchial epithelia cells activation, releasing abundant proinflammatory cytokines such as IL-1β, TNF-α, IL-6 and IL-8, which has been implicated in the pathogenesis of bronchial asthma [34, 35]. Consistent with our results, Garty and his colleagues reported that serum sCD14 expression was significantly increased during acute asthma attacks than that at recovery in asthma patients [19].The sCD14 expression level was significantly increased in bronchoalveolar lavage fluid (BALF) 18 h or 24 h after allergen provocation [18]. Another study also showed that elevated cord blood CD14 level was a predictor of wheeze and prolonged cough in young children [36]. It is suggested that plasma sCD14 level might be elevated during acute asthma attacks in adults and children. However, some other studies showed no association of sCD14 levels with asthma as measured in serum or BALF [37, 38]. These contradictory results are more likely due to the following possible reasons. Firstly, there are various subtypes of asthma defined by distinct pathophysiological mechanisms [39]. In neutrophilic and eosinophilic asthma phenotypes, the level of sCD14 in BALF was obviously decreased with the increasing numbers of neutrophils or eosinophils during acute asthma attacks [40]. Secondly, the discrepancies may be explained by the different stages of asthma patients in these study populations. sCD14 has been considered as an acute phase protein which might be higher elevated in acute attack period than that in remission or stable phases [41]. Thirdly, genetic factors may also have impact on sCD14 expression. Many published papers have reported that TT homozygotes of the CD14 gene had significantly higher density of CD14 receptor on blood monocytes and level of sCD14 in serum when compared with those with a CT or CC genotype [13, 42, 43].

The pathology of adult asthma is characterized by mucus hyperplasia and infiltration of inflammatory cells, among which Th2-cell-dependent inflammation, eosinophils, and neutrophils predominate [39]. Th2-cell derived cytokines including IL-4, IL-5, and IL-13 could contribute to long-term airway hyper-responsiveness and smooth muscle growth, which have significant impact on the decline of lung function and the development of asthma severity [44]. Moreover, both IL-4 and IL-13 have been shown to reduce shedding of CD14 or sCD14 release on a transcriptional level in monocytes and macrophages [45, 46]. These could help to explain our results that plasma sCD14 level was inversely correlated with the probability of FEV_1_/FVC < 75% and the severity of adult asthma. During acute asthma in children, a negative association was also found between asthma severity and plasma sCD14 expression [20]. Thus, it is suggested that the expression of plasma sCD14 could be gradually reduced with the increasing severity of asthma. In addition, it is demonstrated that sCD14 has dual functions in regulation of immune and inflammatory responses. Several studies have indicated that sCD14 plays a critical role in promoting Th1-cell response, but inhibiting the Th2-cell response [12, 13, 47]. Serum sCD14 levels was positively and negatively associated with IFN-γ and IL-4 responses, respectively [13]. Rey Nores and his colleagues reported that sCD14 acts as a negative regulator of human T lymphocyte activation and function by inhibiting T cell proliferation and IL-4 production [16]. More recently, the same authors demonstrated that sCD14 interacted with B cells could suppress IL-6 and IgE production [17]. sCD14 is also thought to have anti-inflammatory effect by transferring LPS into plasma lipoproteins to neutralize its biological activity [48, 49]. Therefore, sCD14 could be considered as an important anti-inflammatory molecule in the process of innate and adaptive immune response, which has a protective effect in the development of asthma. It is supported that plasma sCD14 level might have a predictive effect on the severity of asthma in adults.

To the best of our knowledge, this is the first study that has investigated the association of plasma sCD14 level with the severity of adult asthma in a relatively large population. Moreover, we adjusted a variety of potential lifestyle and environmental confounders by using multivariate logistic regression models, which have potential influences on the level of sCD14 expression. However, there are three limitations in our study. Firstly, asthma is a kind of respiratory disease, but sCD14 expression in plasma is more likely to reflect systemic inflammatory response, rather than specific pulmonary inflammatory response. Therefore, it is necessary to collect some specific samples of respiratory system such as sputum to further demonstrate the relationship between sCD14 expression and the severity of asthma in the future studies. Secondly, the number of severe persistent asthma subgroup is relatively small in present study. Although plasma sCD14 level is significantly negative associated with severe persistent asthma, further study is still needed to explore whether it is similar in a larger population. Thirdly, although allergen challenge has also positive effect on sCD14 expression, we did not analyze the influence of allergy on the association between plasma sCD14 level and the severity of adult asthma. Because there were only 9.12% asthma patients caused by atopy in this study.

## Conclusions

In conclusion, we found that plasma sCD14 level is negatively associated with the severity of adult asthma, suggesting a protective role for sCD14 in the development of asthma in adults. And it is also indicated that plasma sCD14 level might be a potential biomarker in prediction of asthma severity in adults.

## Additional file

Additional file 1: **Table S1.** The Severity Scale of asthma patients in the Guidelines for Prevention and Treatment of Bronchial Asthma in China. (DOC 37 kb)

## Abbreviations

%PRED FEV1: Percent of predicted FEV1
%PRED FVC: Percent of predicted FVC
AUC: Area under the curve
BALF: Bronchoalveolar lavage fluid
BMI: Body mass index
CI: Confidence interval
EDTA: Ethylenediaminetetraacetic acid
ELISA: Enzyme-linked immunosorbent assay
FEV1: forced expiratory volume in one second
FVC: Forced vital capacity
IQRs: Interquartile ranges
LPS: Lipopolysaccharides
mCD14: Membrane-bound CD14
OR: Odd ratio
PEF: Peak expiratory flow
ROC: Receiver operating characteristic
sCD14: soluble CD14
