# Supplementary material for: Association of plasma soluble CD14 level with asthma severity in adults: a case control study in China
Source: Respir Res. 2019 Jan 28;20:19. doi: 10.1186/s12931-019-0987-0 (PMC6348646; doi:10.1186/s12931-019-0987-0)
Supplement: Supplementary file 1 — Table S1. The Severity Scale of asthma patients in the Guidelines for Prevention and Treatment of Bronchial Asthma in China. (DOC 37 kb) [file 12931_2019_987_MOESM1_ESM.doc]

**Association of plasma soluble CD14 level with asthma severity in adults: A case control study in China**

Ting Zhou^1,2^, Xiji Huang^3,4,5^, Jixuan Ma^3,4^, Yun Zhou^3,4^, Yuewei Liu^5^, Lili Xiao^3,4^, Jing Yuan^3,4^, Jungang Xie^6^, Weihong Chen^3,4*^

**Affiliations:**

^1^Department of Occupational and Environmental Health, School of Public Health, Medical College, Wuhan University of Science and Technology, Wuhan, Hubei 430065, China

^2^Hubei Province Key Laboratory of Occupational Hazard Identification and Control, Wuhan University of Science and Technology, Wuhan, Hubei 430065, China

^3^Department of Occupational & Environmental Health, School of Public Health, Tongji Medical College, Huazhong University of Science and Technology, Wuhan, Hubei 430030, China

^4^Key Laboratory of Environment and Health, Ministry of Education & Ministry of Environmental Protection, and State Key Laboratory of Environmental Health (Incubating), School of Public Health, Tongji Medical College, Huazhong University of Science and Technology, Wuhan, Hubei 430030, China

^5^Hubei Center for Disease Control and Prevention, Wuhan, Hubei 430079, China

^6^Department of Respiratory and Critical Care Medicine, Tongji Hospital, Tongji Medical College, Huazhong University of Science and Technology, Wuhan, Hubei 430030, China

**^*^Corresponding author:**

Dr. Weihong Chen

Department of Occupational and Environmental Health,

School of Public Health, Tongji Medical College,

Huazhong University of Science and Technology,

13 Hangkong Road, Wuhan, Hubei, 430030, China

E-mail: [wchen@mails.tjmu.edu.cn](mailto:wchen@mails.tjmu.edu.cn)

Supplementary Table S1 The Severity Scale of asthma patients in the Guidelines for Prevention and Treatment of Bronchial Asthma in China

| Grades | The clinical manifestations |
| --- | --- |
| Intermittent asthma  (Grade 1) | Symptoms less than once a week;  Transient occurrence;  Symptoms at night ≤ 2 times per month;  %PRED FEV_1_ ≥80% or percent of predicted PEF (%PRED PEF%) ≥ 80% and PEF rate variation (PEFRv) <20%. |
|  |  |
| Mild persistent asthma  (Grade 2) | Symptoms more than or equal to once a week but less than once a day;  Might affect activity and sleep;  Symptoms at night > 2 times per month but less than once a week;  %PRED FEV_1_ ≥80% or %PRED PEF ≥ 80% and PEFRv (20%~30%). |
| Moderate persistent asthma  (Grade 3) | Daily symptoms;  Affect activity and sleep;  Symptoms at night ≥ once a week;  %PRED FEV_1_ (60%~79%) or %PRED PEF (60%~79%) and PEFRv >30%. |
|  |  |
| Severe persistent asthma  (Grade 4) | Daily symptoms;  Frequent occurrence;  Symptoms at night frequently;  Physical activity limited  %PRED FEV_1_ <60% or %PRED PEF < 60% and PEFRv > 30%). |
